# Prognostic Factors for Severe-to-Fatal Post-Endoscopic Retrograde Cholangiopancreatography Pancreatitis: A Multicenter Prospective Cohort Study

**DOI:** 10.3390/jcm13041135

**Published:** 2024-02-17

**Authors:** Kazuya Matsumoto, Hisashi Noma, Koichi Fujita, Takeshi Tomoda, Takumi Onoyama, Keiji Hanada, Akihito Okazaki, Ken Hirao, Daisuke Goto, Ichiro Moriyama, Yoshinori Kushiyama, Mamoru Takenaka, Toru Maruo, Hisakazu Matsumoto, Masanori Asada, Hiroko Nebiki, Toshihiro Katayama, Takashi Kawamura, Akira Kurita, Toshiharu Ueki, Masahiro Tsujimae, Tokuhiro Matsubara, Satoshi Yamada, Takashi Tamura, Saiko Marui, Akira Mitoro, Hajime Isomoto, Shujiro Yazumi, Hirofumi Kawamoto

**Affiliations:** 1 Division of Gastroenterology and Nephrology, Faculty of Medicine, Tottori University, Tottori 683-8504, Japan; golf4to@yahoo.co.jp (T.O.); isomoto@tottori-u.ac.jp (H.I.); 2 Irisawa Medical Clinic, Matsue 690-0025, Japan; 3 Department of Data Science, The Institute of Statistical Mathematics, Tachikawa 190-8562, Japan; noma@ism.ac.jp; 4 Department of Gastroenterology and Hepatology, Yodogawa Christian Hospital, Osaka 533-0024, Japan; 5 First Research Department, Medical Research Institute, Kitano Hospital, PIIF Tazuke-Kofukai, Osaka 530-8480, Japan; 6 Department of Gastroenterology and Hepatology, Okayama University Graduate School of Medicine, Dentistry and Pharmaceutical Sciences, Okayama 700-8558, Japan; tomotake79@yahoo.co.jp; 7 Department of Gastroenterology, Onomichi General Hospital, Onomichi, Hiroshima 722-8508, Japan; k.hanada@onomichi-gh.jp; 8 Department of Gastroenterology, Hiroshima Atomic Bomb Survivor Hospital, Hiroshima 730-0052, Japan; ak.from.coast@gmail.com; 9 Department of Internal Medicine, Hiroshima City Hiroshima Citizens Hospital, Hiroshima 730-8518, Japan; dr_ken_hirao@yahoo.co.jp; 10 Department of Internal Medicine, Tottori Red Cross Hospital, Tottori 680-8517, Japan; bluequeel@yahoo.co.jp; 11 Department of Hematology/Oncology, Shimane University Hospital, Innovative Cancer Center, Izumo 693-8501, Japan; ichrmyrm@gmail.com; 12 Department of Gastroenterology, Matsue Red Cross Hospital, Matsue 690-8506, Japan; kushiyama_yoshinori@matsue.jrc.or.jp; 13 Department of Gastroenterology and Hepatology, Kinki University, Osaka 589-8511, Japan; mamoxyo45@gmail.com; 14 Department of Gastroenterology, Fukuoka University Chikushi Hospital, Fukuoka 814-0180, Japan; maruo@fukuoka-u.ac.jp (T.M.); tosiueki@fukuoka-u.ac.jp (T.U.); 15 Department of Gastroenterology, Japan Red Cross Hospital Wakayama Medical Center, Wakayama 640-8558, Japan; ds00308908@yahoo.co.jp; 16 Department of Gastroenterology and Hepatology, Japan Red Cross Osaka Hospital, Osaka 543-8555, Japan; m-asada@osaka-med.jrc.or.jp; 17 Department of Gastroenterology, Osaka City General Hospital, Osaka 534-0021, Japan; hironebi@gmail.com; 18 Help Center of Medical Research, Medical Research Institute, Kitano Hospital, PIIF Tazuke-Kofukai, Osaka 530-8480, Japan; toshiro_katayama@morinomiya-u.ac.jp; 19 Department of Preventive Services, School of Public Health, Graduate School of Medicine, Kyoto University, Kyoto 606-8303, Japan; kawamura.takashi.6u@kyoto-u.jp; 20 Kitano Hospital, Tazuke-Kofukai Medical Research Institute, Kyoto 530-8480, Japan; kuritaaki1976@gmail.com; 21 Department of Gastroenterology, Osaka Saiseikai Nakatsu Hospital, Osaka 530-0012, Japan; kofauthipvkn@yahoo.co.jp; 22 Department of Gastroenterology, Toyonaka Municipal Hospital, Toyonaka, Osaka 560-8565, Japan; tokumax0918@gmail.com; 23 Department of Gastroenterology, Kobe City Medical Center West Hospital, Kobe 650-0047, Japan; yamadasa@kuhp.kyoto-u.ac.jp; 24 Second Department of Internal Medicine, Wakayama Medical University, Wakayama 641-8510, Japan; ttakashi@wakayama-med.ac.jp; 25 Department of Gastroenterology and Hepatology, Kyoto University Graduate School of Medicine, Kyoto 606-8507, Japan; smarui@kuhp.kyoto-u.ac.jp; 26 Department of Gastroenterology, Nara Medical University, Nara 634-8522, Japan; mitoroak@naramed-u.ac.jp; 27 Department of Gastroenterology and Hepatology, Medical Research Institute, Kitano Hospital, PIIF Tazuke-Kofukai, Osaka 530-8480, Japan; s-yazumi@kitano-hp.or.jp; 28 General Internal Medicine 2, Kawasaki Medical School General Medical Center, Okayama 701-0192, Japan; hirofumi.kawamoto@gmail.com

**Keywords:** anti-inflammatory agents, cholangiopancreatography, endoscopic, endoscopic retrograde, non-steroidal, pancreatitis, prognostic factor, sphincterotomy

## Abstract

The prognostic factors associated with severe-to-fatal post-endoscopic retrograde cholangiopancreatography (ERCP) pancreatitis (PEP) remain unclear despite the extensive number of studies on PEP. In total, 3739 ERCP patients with biliary disease with an intact papilla and indicated for ERCP were prospectively enrolled at 36 centers from April 2017 to March 2018. Those with acute pancreatitis diagnosed before ERCP, altered gastrointestinal anatomy, and an American Society of Anesthesiologists (ASA) physical status > 4 were excluded. Univariate and multivariate logistic regression analyses were performed on patient-related factors, operator-related factors, procedure-related factors, and preventive measures to identify potential prognostic factors for severe-to-fatal PEP. Multivariate analyses revealed pancreatic guidewire-assisted biliary cannulation (OR 13.59, 95% CI 4.21–43.83, *p* < 0.001), post-ERCP non-steroidal anti-inflammatory drug (NSAID) administration (OR 11.54, 95% CI 3.83–34.81, *p* < 0.001), and previous pancreatitis (OR 6.94, 95% CI 1.45–33.33, *p* = 0.015) as significant risk factors for severe-to-fatal PEP. Preventive measures included endoscopic biliary sphincterotomy (EST; OR 0.29, 95% CI, 0.11–0.79, *p* = 0.015) and prophylactic pancreatic stents (PPSs; OR 0.11, 95% CI, 0.01–0.87, *p* = 0.036). In biliary ERCP, pancreatic guidewire-assisted biliary cannulation, NSAID administration after ERCP, and previous pancreatitis were risk factors for severe-to-fatal PEP, whereas EST and PPS were significant preventive measures for severe-to-fatal PEP.

## 1. Introduction

Endoscopic retrograde cholangiopancreatography (ERCP) is an essential examination and treatment procedure for biliary and pancreatic diseases. However, ERCP-related procedures have a high risk of adverse events (AEs) such as post-ERCP pancreatitis (PEP), bleeding, perforation, and cholangitis. PEP is the most important complication because it can be fatal in severe cases. Although the frequency of PEP has been reported as 1.6%–15%, recent systematic surveys have reported an incidence of 3.47% (95% confidence interval [CI]: 3.19–3.75) and a fatality rate of 0.11% [1].

Although several reports on the risk factors for PEP are mostly based on retrospective studies, detailed investigations on the best predictive factors are lacking [2,3]. For example, the definition of difficulty in the deep cannulation of the bile duct, which is strongly associated with PEP, has not been unified among past reports, and only a few detailed studies exist on the relationship between bile duct cannulation and the incidence of PEP [4]. Endoscopic retrograde pancreatography (ERP) is a typical risk factor for PEP. Although the clinical significance of ERP differs between ERCP for the biliary tract and ERCP for the pancreatic duct, few studies are focusing exclusively on biliary ERCP. The relationship between PEP and the insertion of a guide wire into the pancreatic duct and the degree of pancreatography has not been fully elucidated.

Although a meta-analysis of randomized controlled trials (RCTs) showed that prophylactic pancreatic stents (PPSs) decreased the incidence of PEP (odds ratio [OR] 0.22; 95% CI, 0.12–0.38; *p* < 0.01) [5], criteria for the high risk of PEP have not been established. In clinical practice, PPSs are indicated for reducing the risk of PEP; the PPS insertion procedure provides easy access to the pancreatic duct, is easy to perform, and facilitates prophylactic pancreatic drainage, which contributes to suppressing PEP. Careful patient selection for PPS insertion is thus useful and required.

The pre-treatment administration of high-dose non-steroidal anti-inflammatory drugs (NSAIDs) is useful for preventing PEP, with a risk ratio of 0.36 (95% CI, 0.22–0.60) [6]. However, the reported administered dose is double the maximum recommended dose in Japan, and side effects such as lower blood pressure, liver dysfunction, gastrointestinal ulcer, and renal damage are concerning; therefore, NSAID administration in such cases is not widely used in Japan.

While many studies have focused on the risk factors for and prevention of PEP, none have been comprehensive, and as PEP is a multifactorial disease, several unsolved problems remain. In particular, severe-to-fatal PEP is the most serious manifestation of PEP, but studies on the risk factors according to disease severity are lacking. We have previously prospectively established a multi-institutional database on the incidents related to biliary ERCP and have reported on the risk factors and preventive measures for PEP in particular [7,8]. In the previous study, 3739 ERCP cases were enrolled among 16,032 ERCP procedures performed at 36 centers. In the current study, we used the same database to analyze the risk factors of severe-to-fatal PEP due to biliary ERCP.

## 2. Materials and Methods

### 2.1. Patient Eligibility

Patients with biliary disease with an intact papilla and significant biliary endotherapeutic experience were prospectively enrolled at 36 centers from April 2017 to March 2018. The inclusion criteria were as follows: intact papilla, indicated for trans-papillary ERCP only in the biliary tract, and no history of pancreatic surgery; age limitations were not imposed. An intact papilla was defined in cases where any treatment for the papilla, such as endoscopic biliary sphincterotomy (EST), balloon dilatation, precut sphincterotomy, or endoscopic treatment on the pancreatic duct or biliary tract, had not been performed. We excluded all patients who exhibited acute pancreatitis diagnosed before ERCP, with an American Society of Anesthesiologists (ASA) physical status ≥ 4, and altered gastrointestinal anatomy and those deemed inappropriate for this study.

### 2.2. Exposure Factors

The selection of risk factors was determined based on a previous study [7,8]. Regarding the suspicion of sphincter of Oddi disorders (SODs), in principle, Rome IV criteria were applied [9]. However, in the case of clinical suspicion, a “suspected SOD” was attributed even if the Rome IV criteria were not satisfied. In the case of bile duct stenosis, the maximum diameter (mm) of the extrahepatic bile duct in the image before ERCP was the diameter of the extrahepatic bile duct on the papillary side of the stenosis. When the extrahepatic bile duct collapsed, and it was difficult to measure, it was defined as “unmeasurable (non-dilated)”. Cholangitis was diagnosed if the case satisfied or was suspected of satisfying the Tokyo Guidelines 2013 criteria [10].

A pancreatic duct obstruction in the pancreatic head was defined when an interruption of the main pancreatic duct in the pancreatic head and a dilation of the pancreatic duct distal to the disruption were observed on imaging. The time required for deep cannulation of the bile duct was the time from the start of the cannulation trial to the deep cannulation of the bile duct after reaching the duodenal papilla; in the case of unsuccessful deep cannulation of the bile duct, this time was defined as the time from the start of the cannulation trial to the end of the examination. The criteria for the use of NSAIDs, route of administration, and criteria for insertion of pancreatic duct stents were left to the policy of each facility.

PEP was defined based on the presence of the following two items: [1] new abdominal pain or enhancement of abdominal pain lasting for 24 h or more after ERCP, and [2] an increase in serum pancreatic enzyme levels of more than three times the standard value after 24 h [11,12,13,14]. The severity of PEP was classified based on the American Society for Gastrointestinal Endoscopy severity grading system [14]. The fasting period was used as an index to assess the prolongation of hospital stay.

### 2.3. Procedure

In this study, the device used for ERCP was not specified. Procedures were carried out under conscious sedation.

### 2.4. Ethical Considerations

This study was carried out after registering the outline of this study in the University Hospital Medical Information Network Clinical Trials Registry (UMIN-CTR, UMIN ID 000024820). As this was a cohort study that did not involve invasive procedures and tissue samples were not collected, we did not obtain informed consent from the patients. An opt-out format was adopted; the study details were announced on a bulletin board, and the opportunity to refuse participation was provided.

### 2.5. Data Collection

A common case report form (CRF) was created for the study. Clinical data recorded on the CRF were prospectively accumulated in the database of each institution and integrated by the study group. Eligibility for this study was determined before ERCP, and ERCP information was recorded immediately after ERCP. In addition, incidents of AEs were assessed 1 week after ERCP, and patient outcomes were assessed one month later.

### 2.6. Primary Objective and Predictor Variables

The primary objective of the study was the analysis of the prognostic factors for severe-to-fatal PEP. These factors were identified by comparing the PEP group with the PEP non-onset group (which was defined as the control group).

### 2.7. Statistical Analysis 

Categorical data were compared using Fisher’s exact test, and continuous variables were compared using the Student’s *t*-test. Univariate and multivariate logistic regression analyses were performed on patient-related factors, operator-related factors, procedure-related factors, and preventive measures to explore the predictors of PEP. Explanatory variables in the multivariate models were selected using forward stepwise selection (*p* < 0.20). In case of missing data, the multiple imputation by chained equations was applied [15]. Statistical analyses were performed using R version 3.5.1 (R Foundation for Statistical Computing, Vienna, Austria).

## 3. Results

### Patient Characteristics and Demographics

The database includes a total of 16,032 ERCP procedures performed at 36 centers; a total of 3739 patients were enrolled in the study. The average patient age was 72.5 years, and 43.7% of the patients were women. The overall success rate of bile duct cannulation was 96.9%. The incidence rate of PEP was 6.9% (258 cases, comprising 201 mild, 39 moderate, 17 severe, and 1 fatal case). The dosage of NSAIDs was as follows: 12.5 mg, *n* = 1; 25 mg, *n* = 253; 50 mg, *n* = 359; and 100 mg, *n* = 2.

Table 1 summarizes the comparison of PEP grades among the various factors. A significant difference was observed among the study groups for the following factors: female sex and age <50 years, ASA status ≥ 3, previous pancreatitis, normal serum bilirubin level (T-bil <1.2 mg/dL), hyperamylasemia before ERCP (pre_amylase level ≥ 130 IU/mL), pancreatic guidewire-assisted biliary cannulation, precut sphincterotomy, difficulty in cannulation for >10 min, pancreatic contrast injection, guidewire insertion for the pancreatic duct, bile duct tissue sampling (brushing cytology), bile duct tissue sampling (biopsy), bile duct-intraductal ultrasonography, PPS use, endoscopic nasopancreatic drainage, total procedure time, procedure time > 60 min, and rectal administration of NSAIDs after ERCP.

Table 2 presents the significant risk factors and preventive measures for severe-to-fatal PEP compared to the control, identified based on the univariate analysis. We confirmed a significant difference in the rectal administration of NSAIDs after ERCP, EST, previous pancreatitis, pancreatic guidewire-assisted biliary cannulation, guidewire insertion for the pancreatic duct, and difficulty in cannulation for >10 min. 

The results of the multivariate analyses (Table 2) revealed that the following were significant risk factors for severe-to-fatal PEP (*p* < 0.05): pancreatic guidewire-assisted biliary cannulation (OR 13.59; 95% CI, 4.21–43.83; *p* < 0.001), rectal administration of NSAIDs after ERCP (OR 11.54; 95% CI, 3.83–34.81; *p* < 0.001), and previous pancreatitis (OR 6.94; 95% CI, 1.45–33.33; *p* = 0.015). The analysis showed that the significant preventive measures for severe-to-fatal PEP were EST (OR 0.29; 95% CI, 0.11–0.79; *p* = 0.015) and PPSs (OR 0.11; 95% CI, 0.01–0.87; *p* = 0.036).

We present a case of extrahepatic bile duct cancer with fatal PEP.

In an 83-year-old woman, biliary ERCP was performed for cholangitis associated with extrahepatic bile duct cancer. No history of acute pancreatitis, no NSAIDs before and after ERCP. Canulation was started using the conventional method and changed to wire-guided canulation due to difficulty in insertion for the bile duct. After brushing cytology, EBS with a 7Fr plastic stent was performed. EST and EPS were not carried out. The next day, she had abdominal pain and increased amylase levels (1856 IU/mL) and was diagnosed with severe pancreatitis by imaging. She subsequently died without her symptoms improving.

## 4. Discussion

In this study, analysis of data from 3739 patients with biliary disease with an intact papilla who underwent ERCP showed that pancreatic guidewire-assisted biliary cannulation, administration of NSAIDs after ERCP, and previous pancreatitis were risk factors and EST and PPSs were significantly preventive for severe-to-fatal PEP. As both pancreatic guidewire-assisted biliary cannulation and previous pancreatitis were identified as risk factors, and as pancreatic guidewire-assisted biliary cannulation is usually selected for patients with difficulty in deep bile duct cannulation, it is highly likely that papilledema (generally regarded as a risk factor for PEP) had already occurred at the stage when this mode of biliary cannulation was selected [13]. Papilledema leads to the stasis of pancreatic juice and increases intrapancreatic duct and pancreatic tissue pressure, which ultimately causes PEP [16]. Furthermore, in pancreatic guidewire-assisted biliary cannulation, indwelling the guidewire too long along the main pancreatic duct or inserting it into the branch pancreatic duct can damage the main pancreatic duct and its branches. Co-occurrence of papilledema and guidewire-induced pancreatic duct injury may predispose patients to severe PEP. In addition, although the reason for a history of pancreatitis is not clear, trypsin and PLA2 are easily activated in such patients [17]; therefore, it is possible that they have a predisposition to developing acute pancreatitis.

EST and PPSs were identified as significant preventive measures for severe-to-fatal PEP. We speculate that EST suppresses the development of severe-to-fatal PEP by reducing pancreatic juice stasis to some extent. We believe that PPSs contribute to preventing papilledema due to ERCP and related procedures, associated pancreatic juice stasis, and elevation of pancreatic duct pressure, thereby reducing the risk of severe PEP. 

NSAID administration after ERCP was confirmed as a risk factor for PEP. Therefore, it is possible that moderate-to-severe PEP may have been caused by postoperative NSAID administration (possibly based on the experience of the operator). Additionally, most of the NSAID doses used in this study were between 25 and 50 mg, which may explain the observation that the incidence of PEP was higher than that in previous reports [6]. In addition, in this study, it is possible that NSAIDs were administered after ERCP to cases that the endoscopists determined to be at risk of developing severe PEP. However, a meta-analysis of four RCTs on the inhibitory effect of NSAIDs on severe-to-fatal PEP [18,19,20,21] revealed that no significant inhibitory effect was observed [22]; this is consistent with the finding of the current study that prophylactic NSAID use after ERCP cannot prevent the onset of PEP of any severity. Therefore, we propose that NSAID administration after ERCP should be avoided if the NSAID dose is less than that used in previous studies. The above discussion indicates the importance of the need for further research into the mechanism underlying the effects of NSAIDs in such patients.

Other factors that have been reported to be associated with PEP include examination time, pancreatography, bile duct biopsy, age, epinephrine dispersal, endoscopic nasobiliary drainage, facility size, and preoperative T.bil value [2,3,4,23,24]. In our study, all of these were factors associated with mild PEP. In biliary ERCP, it is important to avoid the above-mentioned factors associated with severe-to-fatal PEP; thus, it is important to consider the priority of each factor when performing treatment. In a previous report, endoscopic papillary balloon dilatation (EPBD) was a risk factor for PEP [25]; however, EPBD was not a risk factor for even mild PEP in this study. A subsequent report mentioned that the development of PEP was reduced by manipulating the dilatation time and balloon diameter [26,27]. We speculate that EPBD was not identified as a risk factor for PEP in this study because, in this study, the above-mentioned procedure was followed.

In this study, a trainee’s prior performance of ERCP was not a risk factor for PEP. Regarding this factor, it is necessary to consider other ERCP-related complications and the general condition of the patient, but it is considered useful information at the training facility.

This study has some limitations. First, the extent of an incision during EST and the diameter of the stent during PPS use were not examined in this study; these factors need to be considered in future studies. Second, as EPBD and endoscopic papillary large balloon dilatation (EPLBD) are similar procedural factors, they were integrated into a single variable in the multivariate analysis. Therefore, the differences between EPBD and EPLBD were not examined in the analysis.

Endoscopic ultrasound (EUS)-guided cholangioscopic procedures have recently been performed as alternatives to ERCP-related procedures. Such procedures are associated with a lower risk of PEP than ERCP-related procedures [28]. Thus, as a history of pancreatitis was found to be associated with severe-to-fatal PEP in this study, EUS-guided treatment may be given priority over ERCP.

In summary, we found that in biliary ERCP, pancreatic guidewire-assisted biliary cannulation and previous pancreatitis were risk factors for severe-to-fatal PEP, whereas EST and PPSs were identified as significant preventive measures for this condition. For this patient population, this information will aid in selecting patients for ERCP and deciding on prophylactic measures.

## Figures and Tables

**Table 1:** Comparison of post-endoscopic retrograde cholangiopancreatography pancreatitis (PEP) grades among the potential prognostic factors.

Factor	Control (%)	Mild PEP (%)	Moderate PEP (%)	Severe-to-Fatal PEP (%)	*p*-Value
N	3481	201	39	18	
Female sex, <50-year-old	77 (2.2)	10 (5.0)	3 (7.7)	1 (5.6)	0.008
ASA physical status 3	560 (16.1)	18 (9.0)	6 (15.4)	0 (0.0)	0.008
Previous pancreatitis	69 (2.0)	3 (1.5)	2 (5.1)	2 (11.1)	0.038
Suspected sphincter of Oddi dysfunction	31 (0.9)	3 (1.5)	0 (0.0)	0 (0.0)	0.663
Normal serum bilirubin (T-bil < 1.2 mg/dL)	1286 (37.0)	91 (45.3)	22 (57.9)	8 (44.4)	0.006
Pre_amylase ≥ 130 IU/mL	451 (13.4)	16 (8.2)	2 (5.1)	4 (23.5)	0.033
Diameter of extrahepatic bile duct <10 mm	1827 (52.7)	115 (57.5)	25 (64.1)	9 (50.0)	0.284
Cholangitis	1246 (35.8)	51 (25.4)	5 (12.8)	4 (22.2)	<0.001
Trainee status	1694 (48.7)	112 (55.7)	20 (51.3)	11 (61.1)	0.177
Low-volume center (<400 ERCP cases/year)	693 (19.9)	29 (14.4)	11(28.2)	5 (27.8)	0.084
Periampullary diverticulum	859 (25.0)	43 (21.6)	5 (12.8)	3 (16.7)	0.205
Obstruction of the main pancreatic duct at the pancreatic head	347 (10.0)	12 (6.0)	0 (0.0)	1 (2.6)	0.060
Wire-guided cannulation first	776 (22.3)	46 (22.9)	15 (38.5)	4 (22.2)	0.137
Pancreatic guidewire-assisted biliary cannulation	796 (22.9)	88 (43.8)	19 (48.7)	12 (66.7)	<0.001
Precut sphincterotomy	182 (5.2)	21 (10.4)	1 (2.6)	2 (11.1)	0.012
Endoscopic biliary sphincterotomy	2219 (63.8)	121 (60.2)	21 (53.8)	7 (38.9)	0.064
Endoscopic papillary balloon dilatation	135 (3.9)	5 (2.5)	2 (11.1)	3 (7.7)	0.111
Endoscopic papillary large balloon dilatation	117 (3.4)	4 (2.0)	0 (0.0)	1 (2.6)	0.794
Unsuccessful biliary cannulation	103 (3.0)	11 (5.5)	1 (2.6)	0 (0.0)	0.23
Difficulty in cannulation for >10 min	1135 (32.8)	107 (53.5)	22 (56.4)	12 (66.7)	<0.001
Pancreatic contrast injection	1326 (38.1)	126 (62.7)	18 (46.2)	10 (55.6)	<0.001
Guidewire insertion for pancreatic duct	1051 (30.2)	119 (59.2)	21 (53.8)	14 (77.8)	<0.001
Endoscopic biliary stenting (plastic stent)	1476 (42.4)	94 (46.8)	19 (48.7)	7 (38.9)	0.529
Endoscopic nasobiliary drainage	676 (19.4)	30 (14.9)	9 (23.1)	4 (22.2)	0.35
Endoscopic nasogallbladder drainage or endoscopic gallbladder stenting	39 (1.1)	2 (1.0)	0 (0.0)	0 (0.0)	1.000
Endoscopic biliary stenting (metal stent)	202 (5.8)	17 (8.5)	2 (5.1)	0 (0.0)	0.37
Extraction of biliary stones	1199 (34.4)	53 (26.4)	12 (30.8)	5 (27.8)	0.108
Bile duct tissue sampling (brushing cytology)	331 (9.5)	27 (13.4)	6 (15.4)	4 (22.2)	0.037
Bile duct tissue sampling (biopsy)	314 (9.0)	31 (15.4)	7 (17.9)	3 (16.7)	0.003
Bile duct-intraductal ultrasonography	325 (9.3)	35 (17.4)	8 (20.5)	0 (0.0)	<0.001
Prophylactic pancreatic stents	333 (9.6)	38 (18.9)	6 (15.4)	1 (5.6)	<0.001
Endoscopic nasopancreatic drainage	13 (0.4)	3 (1.5)	1 (2.6)	0 (0.0)	0.039
Procedure time > 60 min	436 (12.5)	60 (30.0)	11 (28.2)	2 (11.1)	<0.001
Protease inhibitor	2809 (80.7)	164 (81.6)	32 (82.1)	15 (83.3)	0.994
Glyceryl trinitrate	25 (0.7)	1 (0.5)	1 (2.6)	0 (0.0)	0.378
Spraying saline–epinephrine	960 (27.6)	41 (20.4)	7 (17.9)	5 (27.8)	0.080
Isotonic contrast agent	1989 (57.1)	107 (53.2)	18 (46.2)	12 (66.7)	0.297
Rectal administration of NSAIDs before ERCP	354 (10.2)	19 (9.5)	6 (15.4)	3 (16.7)	0.554
Rectal administration of NSAIDs after ERCP	203 (5.8)	19 (9.5)	8 (20.5)	6 (33.3)	<0.001

ASA: American Society of Anesthesiologists; ERCP: post-endoscopic retrograde cholangiopancreatography; NSAIDs: non-steroidal anti-inflammatory drugs.

**Table 2:** Significant risk factors and preventive measures for severe-to-fatal post-endoscopic retrograde cholangiopancreatography pancreatitis (PEP) compared to control as per univariate and multivariate analyses.

Factor	Univariate Analysis			Multivariate Analysis		
	OR	95% CI	*p*-Value	OR	95% CI	*p*-Value
Female sex and <50-year-old	2.60	0.34–19.80	0.356			
ASA physical status 3	0.00	Inestimable	0.989			
Previous pancreatitis	6.18	1.39–27.41	0.017	6.94	1.45–33.33	0.015
Suspected sphincter of Oddi dysfunction	0.00	Inestimable	0.991			
Normal serum bilirubin (T-bil < 1.2 mg/dL)	1.36	0.53 –3.45	0.520			
Pre_amylase ≥ 130 IU/mL	2.14	0.70–6.54	0.180			
Diameter of extrahepatic bile duct <10 mm	0.90	0.36–2.27	0.822			
Cholangitis	0.51	0.17–1.56	0.239			
Trainee status	1.66	0.64–4.29	0.297			
Low-volume center (<400 ERCP cases/year)	1.55	0.55–4.36	0.408			
Periampullary diverticulum	0.60	0.17–2.08	0.419			
Obstruction of the main pancreatic duct at the pancreatic head	0.00	Inestimable	0.987			
Wire-guided cannulation first	1.00	0.33–3.04	0.994			
Pancreatic guidewire-assisted biliary cannulation	6.88	2.57–18.40	<0.001	13.59	4.21–43.83	<0.001
Precut sphincterotomy	2.26	0.52–9.90	0.280			
Endoscopic biliary sphincterotomy	0.36	0.14–0.94	0.036	0.29	0.11–0.79	0.015
Endoscopic papillary balloon dilatation	3.10	0.71–13.62	0134			
Endoscopic papillary large balloon dilatation	0.00	Inestimable	0.988			
Unsuccessful biliary cannulation	0.00	Inestimable	0.989			
Difficulty of cannulation for >10 min	4.08	1.53–10.90	0.005			
Pancreatic contrast injection	2.03	0.80–5.16	0.136			
Guidewire insertion for the pancreatic duct	8.09	2.66–24.65	<0.001	4.83	0.86–27.08	0.073
Endoscopic biliary stenting (plastic stent)	0.85	0.33–2.19	0.731			
Endoscopic nasobiliary drainage	1.18	0.39–3.61	0.766			
Endoscopic biliary stenting (metal stent)	0.00	Inestimable	0.985			
Extraction of biliary stones	0.73	0.26–2.06	0.554			
Bile duct tissue sampling (brushing cytology)	2.72	0.89–8.30	0.080	2.77	0.86–8.88	0.087
Bile duct tissue sampling (biopsy)	2.02	0.58–7.01	0.269			
Bile duct-intraductal ultrasonography	0.00	Inestimable	0.988			
Prophylactic pancreatic stents	0.56	0.07–4.19	0.569	0.11	0.01–0.87	0.036
Endoscopic nasopancreatic drainage	0.00	Inestimable	0.991			
Procedure time > 60 min	0.87	0.20–3.80	0.855			
Protease inhibitor	1.20	0.35–4.15	0.778			
Glyceryl trinitrate	0.00	Inestimable	0.988			
Spraying saline–epinephrine	1.01	0.360–2.84	0.985			
Isotonic contrast agent	0.67	0.25–1.78	0.418			
Rectal administration of NSAID therapy before ERCP	1.76	0.51–6.12	0.373	3.14	0.80–12.31	0.102
Rectal administration of NSAID therapy after ERCP	8.12	3.01–21.86	<0.001	11.54	3.83–34.81	<0.001

CI: confidence interval; ERCP: endoscopic retrograde cholangiopancreatography; NSAID: non-steroidal anti-inflammatory drug; OR: odds ratio.

## Data Availability

The data that support the findings of the study are available upon request from the corresponding author.
